# *HMGCR* genetic variability in Parkinson’s disease in a Spanish cohort: associations with lipid metabolism and early onset

**DOI:** 10.1007/s00415-025-13404-6

**Published:** 2025-10-02

**Authors:** Rafael Díaz-Belloso, Miguel Martín-Bornez, Daniel Macías-García, Sergio García-Díaz, Marta Bonilla-Toribio, Dolores Buiza-Rueda, Rocío Pineda Sánchez, Laura Muñoz-Delgado, Elena Ojeda, Silvia Jesús, Astrid Adarmes-Gómez, Fátima Carrillo, Pablo Mir, Pilar Gómez-Garre

**Affiliations:** 1 Servicio de Neurología, Unidad de Trastornos del MovimientoInstituto de Biomedicina de Sevilla (IBiS)/Hospital Universitario Virgen del Rocío/CSICUniversidad de Sevilla, Avda. Manuel Siurot s/n, 41013 Seville, Spain; 2 Centro de Investigación Biomédica en Red Sobre Enfermedades Neurodegenerativas (CIBERNED), Instituto de Salud Carlos III, Madrid, Spain; 3 Departamento de Medicina, Facultad de Medicina, Universidad de Sevilla, Seville, Spain

**Keywords:** Early onset Parkinson’s disease, Risk, Association, Lipid metabolism, Spanish cohort, Genetic variants

## Abstract

Lipid metabolism has emerged as a key factor in the pathophysiology of Parkinson’s disease (PD). While 3-hydroxy-3-methylglutaryl-coenzyme A reductase (HMGCR), the rate-limiting enzyme in cholesterol biosynthesis, has been implicated in neurodegenerative disorders, such as Alzheimer disease, its role in PD remains unexplored. Given the shared pathological features between these neurodegenerative disorders (such as lysosomal dysfunction and lipid dysregulation) we hypothesized that *HMGCR* genetic variability could influence PD susceptibility and/or phenotypic expression, particularly in early-onset cases (EOPD)**.** Therefore, we performed targeted sequencing of *HMGCR* in 1162 Spanish PD patients and analyzed associations with PD risk or age at onset. Replication was attempted in 436 PD cases from Parkinson’s Progression Markers Initiative (PPMI). We identified 21 *HMGCR* variants, including a likely pathogenic variant affecting the splice site of exon 4 (c.278-1G > A) in a patient with early-onset PD (EOPD), rapid progression, and severe dyslipidemia. The rs5908 variant was significantly associated with EOPD in our cohort (OR = 2.22, p = 0.025). Further analysis revealed rs5908 is in linkage disequilibrium with rs115169875, an intronic variant with putative regulatory impact. Our findings nominate *HMGCR* as a candidate gene for PD development, particularly in early-onset cases. The metabolic profile of the c.278-1G > A carrier parallels *GBA*-associated PD, suggesting convergent lipid-lysosomal pathways in neurodegeneration. While cohort differences highlight population-specific genetic effects, these findings underscore the importance of lipid pathways in PD and the need to explore *HMGCR*’s role in PD subtypes with metabolic comorbidity.

**Supplementary Information:**

The online version contains supplementary material available at 10.1007/s00415-025-13404-6.

## Introduction

Parkinson’s disease (PD) is the most common neurodegenerative movement disorder [23]. Its primary pathological hallmark is the progressive loss of dopaminergic neurons in the substantia nigra pars compacta, and the accumulation of misfolded alpha-synuclein (a-Syn) in intraneuronal inclusions known as Lewy bodies [3]. While genetic factors contribute significantly to PD pathogenesis, particularly in early-onset PD (EOPD), the interaction between genetic susceptibility and metabolic pathways remains poorly understood.

In the last years, abnormalities in the lipid metabolism and the identification of associated genetic risk factors have been related with mechanisms that may directly or indirectly contribute to PD development. Cholesterol plays an essential role in neuronal physiology, and the consequences of its depletion include impaired synaptic vesicles exocytosis and degeneration of neurotransmission and synapsis [21, 45]. Altered cholesterol metabolism in the brain has been reported in PD patients, and key PD-related genes such as *LRRK2* and *GBA*, have been associated with cholesterol imbalance [15, 21, 29]. In addition, Lewy bodies are enriched with both a-Syn and various lipid species [12, 13], emphasizing a connection between lipid dysregulation and PD pathophysiology.

In recent years, genetic factors affecting lipid metabolism have also been described in other neurodegenerative disorders [25]. The role of cholesterol in neurodegeneration is exemplified by APOE, the principal cholesterol transporter in the brain and the strongest genetic risk factor for Alzheimer’s disease and Lewy body dementia [9, 10, 43]. Nevertheless, despite evidence supporting the involvement of cholesterol at both cerebral and peripheral levels in PD, this relationship is complex and not yet fully understood.

**Fig. 1:**
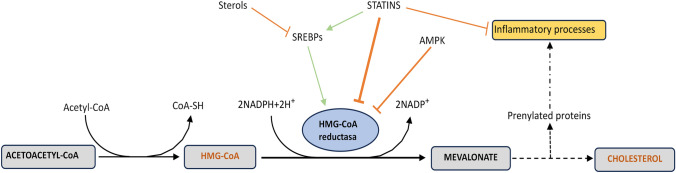
HMGCR in the cholesterol biosynthesis pathway. HMGCR is the target of the statin class of drugs. HMG-CoA: 3-hydroxy-3-methylglutaryl coenzyme A; SREBPs: Sterol regulatory element-binding protein; AMPK: AMP Kinase; Statins competitively inhibit HMG-CoA reductase

In this context, the enzyme 3-Hydroxy-3-Methylglutaryl-CoA reductase (HMGCR), emerges as a candidate for PD susceptibility. HMGCR regulates the rate-limiting step of the cholesterol synthesis [36, 40] and is the therapeutic target of statins (Fig. 1). Previous studies have implicated *HMGCR* in Alzheimer’s disease (AD) as a genetic modifier of risk and cognitive decline, comparable to *APOE*, the main cholesterol transporter in the brain [5, 26]. Although AD and PD are distinct entities, there are overlapping clinical and pathological features of AD and PD (such as dementia common in PD patients and parkinsonism occurring in AD patients) that suggest shared pathogenic mechanisms[7, 46]. However, *HMGCR* remains unexplored in PD.

Based on this rationale, we hypothesized that *HMGCR* genetic variability might influence PD susceptibility or phenotypic expression. Therefore, we assessed the *HMGCR* genetic variability in a well-characterized cohort of patients with PD from southern Spain**.**

## Materials and methods

We conducted a genetic study of *HMGCR* variants in PD using a discovery-replication design.

Genomic DNA was extracted from peripheral whole blood samples following manual and automatic extraction protocols (DNA Isolation Kit for Mammalian Blood, Roche Diagnostics, Mannheim Germany; MagNA Pure LC, Roche Diagnostics). DNA concentration and quality were assessed using a Qubit 2.0 fluorometer and a NanoDrop2000 spectrophotometer (Thermo Scientific™).

### Study cohorts

The discovery cohort included 1162 unrelated PD patients (Table 1) recruited from the Movement Disorders Clinic at Hospital Universitario Virgen del Rocío (HUVR) in Seville (Spain). All participants self-reported European ancestry, underwent extensive clinical phenotyping and met established diagnostic criteria for PD. The PD diagnosis was performed by neurologists specialized in movement disorders according to the United Kingdom Parkinson’s Disease Society Brain Bank criteria [17] for patients recruited up to 2018, and the MDS clinical diagnostic criteria [37] from 2019 onward. A family history of PD was considered positive if patients reported at least one first-degree relative with PD. EOPD was defined as disease onset at age ≤ 50 years. All patients had previously been screened for variations in major PD-related genes (Supplementary Table 1) [18].

**Table 1:** Demographic characteristics of the Spanish PD cohort

	PD cohort				*HMGCR* variation carriers			
	**N**	% Men (M/F)	Mean age ± SD (y)	Mean AAO ± SD (y)	N	% Men (M/F)	Mean age ± SD (y)	Mean AAO ± SD (y)
Total PD	1162	60.0 (697/465)	63.9 ± 11.3	56.1 ± 11.8	91	60.4 (55/36)	61.7 ± 12.2	53.3 ± 12.7
Family PD	157	54.1 (85/72)	64.6 ± 10.7	56.3 ± 12.4	18	55.6 (10/8)	59.3 ± 10.1	50.9 ± 12.1
EOPD	375	63.7 (239/136)	53.1 ± 9.1	42.8 ± 7.3	42	61.9 (26/16)	52.8 ± 9.23	42.6 ± 8.0

*N* number of subjects, *M* male, *F* female, *SD* standard deviation, *y* years, *AAO* age at onset, *EOPD* early onset PD (≤ 50 years)

For replication, we analyzed data from 436 PD patients from the Parkinson’s Progression Markers Initiative (PPMI). This cohort met the following inclusion criteria: white Caucasian ancestry, documented age at onset (AAO), and available genetic data for *HMGCR* gene. Details of the PPMI study design have been published previously and they are accessible at the PPMI website [31, 35].

### Targeted sequencing

We performed targeted genome sequencing (TGS) of all *HMGCR* coding exons (NM_000859.3) and flanking splice regions using the HyperDesign tool (Roche Diagnostics). DNA libraries were prepared with the KAPA HyperPlus kit (Roche Diagnostics) following the manufacturer’s protocols and sequenced on an Illumina NextSeq 500 platform (Illumina, San Diego, CA, USA). Sequencing data were analyzed using Sarek pipeline v3.4.2 [20]. Briefly, it included read quality control with FastQC v0.12.1, reads trimming with Fastp v0.23.4, and aligment of clean reads to the hg38 human reference genome using the Burrows-Wheeler Aligner (BWA v0.7.18). Variant calling was performed with the Genome Analysis Toolkit (GATK v4.5.0), applying stringent filters: minimum read depth ≥ 20, genotype quality ≥ 20, alternative allele ratio ≥ 30%, and a missingness rate ≤ 0.1. Variants were annotated with the Variant Effect Predictor tool [32] and Varsome database [24]. Variants with depth of coverage and genotype quality ≥ 20 were retained. Additional filters included variants in the boundaries of the coding regions extended up to 35 bp from the exon.

### Bioinformatic analysis of variants

Copy number variations (CNVs) in the *HMGCR* gene were analyzed using Expert Variant Interpreter (enGenome, Italy) and four programs: ExomeDepth, ConVaDING, CODEX2, and panelcn.MOPS. Losses were defined by a maximum log2 ratio of − 0.55 and a minimum log2 ratio of 0.4 for gains [38].

The potential effects of the variants were evaluated based on their consequences at the protein level and classified according to the American College of Medical Genetics and Genomics (ACMG) criteria. The impact of variants on splicing was assessed using SpliceAI [22] and Human Splicing Finder (HSF) [8]. For HSF, a differential in the consensus values (CVs) for the wild type and variated sequences of 10% or more was considered significant [8]. A linkage disequilibrium (LD) analysis was conducted to identify correlations of rs5908 with non-coding variants. To evaluate the regulatory potential of variants in LD with rs5908, we used LDpop [1] and HaploReg v.4.2 [41].

### Association analysis

Rare variants (0.001 < MAF < 0.05) were analyzed for associations with PD risk and AAO. For PD risk, allele frequencies in the PD cohort were compared to those of healthy Spanish controls from the Collaborative Spanish Variant Server (CSVS), as well as to non-Finnish European and global populations from gnomAD v4.1, using Fisher’s exact test. For association with AAO, the PD cohort was stratified into EOPD and late onset PD (LOPD), and we performed logistic regression models (allelic, genotypic, dominant, and recessive). The primary models were adjusted for sex and for carrier status of variants in known EOPD-related genes (*PRKN, PINK1, SNCA*) [39]. Statistical analyses were conducted using R v.4.2.2 and gPLINK v2.05. Significance was set at *p* ≤ 0.05 with Bonferroni correction.

### Genetic burden tests analysis

We assessed variant burden for PD and AAO using the Consistent summary Counts based Rare Variant burden test (CoCoRV) [6] and R v.4.2.2, respectively. For burden analyses only synonymous variants were excluded. In addition, only variants with population allele frequency ≤ 0.05 in the non-Finnish European subcohort (N-FE) were included.

## Results

Targeted sequencing of *HMGCR* gene in our discovery cohort of 1,162 PD Spanish patients identified 21 distinct variants, with 91 patients (7.83%) carrying at least one variant. The demographic characteristics of the cohort are summarized in Table 1.

Among the 21 variants detected, there were: 4 intronic, 4 synonymous, 6 missense, 2 in the 3’ UTR region, and 5 affecting splice regions (Table 2). All variants occurred in heterozygous state, except for one homozygous case (c.1912A > G, p.Ile638Val) and four compound heterozygotes, including one EOPD patient (AAO = 41 years) carrying both c.1190-18 T > C and 1912 A > G variants.

**Table 2:** Genetic variants in the *HMGCR* gene detected in a Spanish PD cohort

POS (hg38)	Consequence	Candidate variant	rs	ACMG		AC	AF
				Classification	Criteria		
5:75,342,641	missense	c.36 T > A; p.Phe12Leu	–	VUS	PM2|PP2 |BP4	1	0.0004
5:75,344,244	splice variant	c.278-1G > A	–	LP	PVS1 |PM2	1	0.0004
5:75,344,292	missense	c.325G > C; p.Val109Leu	–	VUS	PM2 |PP2	1	0.0004
5:75,345,539	intron	c.366-35C > T	rs200265078	B/LB	PM2|BP4	2	0.0009
5:75,347,330	intron	c.556 + 21 A > G	rs745659867	B/LB	PM2|BP4	2	0.0009
5:75,350,905	synonymous	c.897G > A; p.Lys299 =	rs756709926	B/LB	BP4 |BP7 |PM2	1	0.0004
5:75,350,917	synonymous	c.909A > G; p.Pro303 =	rs953163444	B/LB	BP4 |BP7 |PM2	1	0.0004
5:75,351,064	splice variant	c.942-4 T > G	rs894240033	B/LB	PM2 |BP4	1	0.0004
5:75,351,256	missense	c.1130G > A; p.Arg377Lys	rs753098010	B/LB	PP2|BP4|PP2	1	0.0004
5:75,351,304	missense	c.1178G > A; p.Arg393Gln	rs376488768	B/LB	PP2|BP4|PP2	1	0.0004
5:75,351,406	intron	c.1190-18 T > C	-	B/LB	PM2|BP4	1	0.0004
5:75,351,434	synonymous	c.1200A > C; p.Ile400 =	rs188984384	B/LB	BP4|BP7|PM2	1	0.0004
5:75,351,467	missense	c.1233C > G; p.Asn411Lys	rs201862213	B/LB	BP4|PP2|PM2	1	0.0004
5:75,356,374	missense	c.1912A > G; p.Ile638Val	rs5908	B/LB	BS1. BS2| BP4| BP6	46	0.0198
5:75,356,406	synonymous	c.1944G > A; p.Gln648 =	rs756807553	B/LB	BP4|BP7|PM2	1	0.0004
5:75,358,854	intron	c.2157 + 32del	rs781750120	B/LB	PM2|BP4	1	0.0004
5:75,359,391	splice variant	c.2299-7 T > A	rs199829764	B/LB	BS1|BS2| BP4	2	0.0009
5:75,359,394	splice variant	c.2299-4C > T	rs1051299124	B/LB	PM2|BP4	1	0.0004
5:75,359,976	splice variant	c.2458-9 T > C	rs376514243	B/LB	PM2|BP4	1	0.0004
5:75,360,376	3 prime UTR	c.*34 T > C	rs377093901	B/LB	BS1|BS2| BP4	6	0.0026
5:75,360,423	3 prime UTR	c.*81A > G	rs144433856	B/LB	BP4|PM2	23	0.0100

Table includes the identification and frequency of each variant, and its classification based on ACMG (American College of Medical Genetics and Genomics) criteria, providing a comprehensive characterization of their potential clinical significance. Variants classified as benign or likely benign according to ACMG criteria are shown together in the table for simplicity. Individual ACMG evidence codes applied to each variant remain unchanged

*POS* position, *UTR* untranslated region, *AC* allele count, *AF* allele frequency, *B* bening, *LB* likely bening, *VUS* variant of uncertain significance, *LP* likely pathogenic

According to the ACMG criteria, most variants were classified as benign, likely benign, or variants of uncertain significance (VUS), except for the splice acceptor variant c.278-1G > A, classified as likely pathogenic. This variant was found in a patient with EOPD (age at onset of 50 years) who developed a tremor-dominant PD form with subsequent hallucinations and cognitive impairment after 14 years of disease progression. This variant showed strong in silico evidence for splicing disruption with a SpliceAI acceptor loss Δ score of 0.99, and HSF analysis revealing loss of the canonical acceptor site (ΔCV = − 31.85%) with activation of a cryptic acceptor site (ΔCV = 53.96%). Unfortunately, a sample of the patient carrying this variant was not available for RNA analysis. The patient was diabetic and exhibited moderate hypercholesterolemia (total cholesterol: 284 mg/dl; HDL-cholesterol: 36 mg/dl; LDL-cholesterol: 158.80 mg/dl; LDL/HDL 4.4) and severe hypertriglyceridemia (501 mg/dL), with no history of statin use. She died at the age of 67 with a score of 4 in the Hoehn & Yahr scale.

All the genetic variants described in our PD cohort were very rare (MAF < 0.001), suggesting that they might represent sporadic occurrences in our population, except for three variants: rs5908 (n = 46, MAF = 0.020), rs144433856 (n = 23; MAF = 0.010) and rs377093901 (n = 6; MAF = 0.003). The rs5908 variant showed a significant association with EOPD under both allelic (OR = 2.22; p = 0.025) and dominant models (OR = 2.19; p = 0.034), after Bonferroni correction, whereas no significant associations were found for rs144433856 or rs377093901.

EOPD prevalence among variant carriers was 47.8% (22/46) for rs5908, 43.5% (10/23) for rs144433856, and 50.0% (3/6) for rs377093901. Linkage disequilibrium analysis revealed perfect correlation (r^2^ = 1) between rs5908 and rs115169875 in the Iberian population. HaploReg analysis indicated that rs115169875 is in a regulatory region potentially affecting transcription factor binding and POL2 interaction.

In the replication analysis using the PPMI cohort, we observed comparable allele frequencies for rs5908 (MAF = 0.019 vs. 0.020 in our Spanish cohort) but no significant association with EOPD. The PPMI cohort differed notably from our Spanish cohort in several aspects (Table 3) such as a lower EOPD prevalence (19.2% vs. 32.3%), higher frequency of carriers of mutations in EOPD-associated genes (PRKN/PINK1/SNCA: 6.2% vs. 1.9%), and different recruitment criteria that may have influenced phenotypic distributions.

**Table 3:** Descriptive features of Spanish (discovery) and PPMI (replication) cohorts tested for association of rare variants and EOPD

Cohort	N	% Men (M)	% EOPD (EOPD)	Mean AAO ± SD (y)	total PRKN, PINK1, SNCA carriers	EOPD PRKN, PINK1, SNCA carriers	Total MAF rs5908	EOPD MAF rs5908
Spanish	1162	60.0% (697)	32.3% (375)	61.7 ± 12.2	1.9% (22)	4.5% (17)	0.020	0.029
PPMI	436	57.8% (252)	19.2% (86)	59.78 ± 10.81	6.2% (27)	23.3% (20)	0.019	0.006

*N* number of subjects, *M* male, *SD* standard deviation, *y* years, *AAO* age at onset, *EOPD* early onset PD (PD onset ≤ 50 years), *AF* Allele frequency

We selected the adjusted (inclusive) model as our primary analysis to maximize power while accounting for the potential confounding effect of known EOPD genes. To assess robustness, as a sensitivity analysis, we repeated the association test but excluding carriers of pathogenic/likely pathogenic *PRKN*, *PINK1* and *SNCA* variants for rs5908. Therefore, the association for rs5908 persisted in our cohort, remaining directionally consistent and statistically significant. However, in the PPMI cohort this exclusion left only one rs5908 carrier, making replication unfeasible due to above mentioned differences in cohort composition (lower EOPD prevalence and higher proportion of known-mutation carriers). Comparison with global reference cohorts covering individuals from diverse ancestries revealed significant differences in allele frequencies of rs144433856. However, no statistically significant differences were found when compared with the local control cohort (Supplementary Table 2).

Finally, rare variant burden analysis revealed no significant association between *HMGCR* variants and either PD risk or AAO in either cohort and copy number variation analysis detected no clinically relevant duplications or deletions in the *HMGCR* gene region across all samples studied.

## Discussion

Our study provides the first comprehensive analysis of *HMGCR* genetic variability in PD, revealing novel insights into the potential role of cholesterol mechanism in PD pathogenesis. We identified 21 rare variants in *HMGCR*, including a likely pathogenic splice-site variant (c.278-1G > A) in a patient with a metabolically complex EOPD, dementia, hallucinations, and atypical disease progression. Furthermore, the coexistence of diabetes and hyperglyceridemia was noted, consistent with metabolic syndrome (MetS) that may be relevant for clinical progression [7, 27, 46] Moreover, although the index patient carrying the c.278-1G > A variant had never received statin therapy, systematic information on statin use was not available for the entire cohort. This represents a limitation, since statins inhibit HMGCR activity and may influence PD risk and progression [36].

Additionally, the rs5908 variant showed a significant association with EOPD in our cohort, though this association was not replicated in the PPMI dataset. In silico analyses strongly suggest a disruption of normal splicing, which could affect HMGCR enzymatic activity and cholesterol biosynthesis. Although experimental validation is required, the potential functional relevance of this variant points to a previously unexplored mechanism linking cholesterol metabolism dysregulation and PD. This is consistent with the emerging evidence indicating that alterations in lipid metabolism homeostasis contribute to neurodegenerative processes [7, 12, 14].

Interestingly, the clinical presentation of the patient carrying the c.278-1G > A variant shares features with GBA-associated PD (GBA-PD), including earlier onset, faster progression, and prominent cognitive symptoms [29]. Although the precise mechanisms linking to PD remain unclear. both GBA and HMGCR disruptions may converge on common pathways related to lysosomal dysfunction and lipid dysregulation. Mutations in GBA1 reduce lysosomal glucocerebrosidase (GCase) levels and activity due to its partial retention in the endoplasmic reticulum, leading to endoplasmic reticulum stress, impaired autophagy, and apoptosis[16]. In GBA-PD, glucocerebrosidase deficiency leads to lipid accumulation, with potential effects on α-Syn oligomerization at the synaptic membrane, which further compromises lysosomal degradation and causes neurotoxicity [11]. Experimental models of GBA1 deficiency consistently show lysosomal cholesterol accumulation, which has been linked to lysosomal degeneration and increased neuronal vulnerability [16, 30]. Analogously, impaired HMGCR activity could alter cholesterol biosynthesis, a condition previously reported in both idiopathic and GBA-PD cases [29, 34]. These observations suggest a pathogenic axis where disrupted lipid metabolism and lysosomal dysfunction could hypothetically contribute to processes such as α-Syn and thereby to Lewy bodies formation, the main pathological hallmark of PD, and neurodegeneration.

Among the variants identified, the rs5908 was the only one showing significant association with EOPD in our discovery cohort. Although this association was not replicated in the PPMI cohort, the lack of replication may reflect differences in cohort composition, such as a lower prevalence of EOPD (19.2% vs. 32.3%), higher frequency of carriers of mutations in major EOPD genes (6.2% vs. 1.9%), and divergent recruitment criteria. These disparities underscore the importance of population-specific genetic architectures when interpreting association studies.

Linkage disequilibrium (LD) analysis revealed that rs5908 is in perfect LD (r^2^ = 1) with rs115169875, a non-coding regulatory variant, predicted to affect multiple transcription factor binding sites and reduce POL2 binding. Although we did not directly measure enzymatic activity for the variants identified, such regulatory disruptions may reduce transcriptional efficiency, leading to insufficient cholesterol biosynthesis, which in turn could increase neuronal susceptibility to stress and degeneration.

Despite the lack of statistically significant associations in rare variant burden testing, our study supports a broader role of HMGCR in neurodegeneration. Several previous studies have linked the rs3846662 G allele with an increased risk of cognitive impairment in PD, while the A allele has shown a protective effect in AD [5, 26, 36, 42]. In addition, rare pathogenic mutations in *HMGCR* have been implicated in limb-girdle muscular dystrophy under a recessive model [33, 44], with functional studies showing that *HMGCR* depletion impairs mitochondrial function and promotes apoptosis [19].

However, only a few studies have investigated the impact of *HMGCR* variants specifically in PD. Mendelian randomization analyses have found no consistent association between reduced LDL-C (low-density lipoprotein-cholesterol) levels due to *HMGCR* variants and PD risk [4, 28]. These discrepancies highlight the complexity of cholesterol’s role in neurodegeneration and the need to distinguish between peripheral lipid levels and central nervous system cholesterol homeostasis, which is independently regulated.

While it remains unclear whether increased or decreased cholesterol levels are beneficial in PD, there is growing consensus that cholesterol availability and lipid homeostasis are important for maintaining neuronal integrity. These factors are key regulators of processes frequently disrupted in neurodegenerative diseases, including neuroinflammation, mitochondrial dysfunction, and impaired protein degradation systems, all pathways implicated in PD pathophysiology [2, 36].

In conclusion, our study identifies HMGCR as a potential contributor to PD pathogenesis, particularly in EOPD. Rare and regulatory variants in *HMGCR*, together with its central role in lipid metabolism, positions it as a valuable target for understanding shared pathogenic pathways in neurodegenerative diseases. Further mechanistic studies, assessment of gene-environment interactions (e.g. statin use), and replication across diverse populations, are needed to confirm these findings. From a clinical perspective, the identification of *HMGCR* variants may help define PD subtypes with metabolic comorbidities and guide the development of targeted, metabolism-informed therapeutic strategies, offering a pathway toward more personalized interventions in PD and related disorders.

## Supplementary Information

Below is the link to the electronic supplementary material.

Supplementary file1 (DOCX 35 KB)

## Data Availability

The datasets generated and analyzed during the current study are available from the corresponding author on reasonable request.
