# Supplementary material for: HMGCR genetic variability in Parkinson’s disease in a Spanish cohort: associations with lipid metabolism and early onset
Source: J Neurol. 2025 Oct 2;272(10):671. doi: 10.1007/s00415-025-13404-6 (PMC12491363; doi:10.1007/s00415-025-13404-6)
Supplement: Supplementary file 1 — Supplementary file1 (DOCX 35 KB) [file 415_2025_13404_MOESM1_ESM.docx]

**Table 1. PD-related genes variants reported by Gómez‐Garre et al. in patients from our cohort with *HMGCR* variants.**

| **ID** | **Sex** | **Age (Y)** | **AAO**  **(Y)** | **Family PD** | **Relative with PD** | ***HMGCR* variant** | **Gene** | **Isoform** | **Loci**  **PARK variant** | **ACMG classification** | **GBA1 variants classification*** | **Zygosity** | **Inheritance** | **Genetic diagnosis** |
| --- | --- | --- | --- | --- | --- | --- | --- | --- | --- | --- | --- | --- | --- | --- |
| IP01227 | M | 56 | 30 | Yes | sister | c.*81A>G | *PARK2* | NM_004562.3 | p.Asn52fs; c.155delA | LP | na | Het | AR | Positive |
|  |  |  |  |  |  |  |  |  | exons 3 and 4 deletion | LP | na | Het |  |  |
| IP01275 | M | 57 | 46 | No | - | c.1912A>G | *PARK2* | NM_004562.3 | p.Asn52fs; c.155delA | LP | na | Het | AR | Unknown |
| IP01809 | F | 68 | 49 | No | - | c.1912A>G | *VPS13C* | NM_020821.3 | p.Gln112fs; c.333dupA | P | na | Het | AR | Unknown |
| IP03511 | F | 47 | 36 | No | - | c.*81A>G | *ATP13A2* | NM_022089.4 | c.477+2T>G | P | na | Het | AR | Unkonwn |
| IP04355 | M | 53 | 45 | Yes | mother | c.942-4T>G | *GBA1* | NM_000157.4 | p.Glu365Lys; c.1093G>A | B | Risk variant | Het | AD/RF | Unknown |
| IP11612 | F | 53 | 31 | Yes | 2 brothers | c.1912A>G | *PARK2* | NM_004562.3 | p.Asn52fs; c.155delA | LP | na | Hom | AR | Positive |
| IP09236 | M | 59 | 46 | No | - | c.*81A>G | *PARK2* | NM_004562.3 | p.Asn52fs; c.155delA | LP | na | Het | AR | Unknown |
| IP13088 | M | 65 | 42 | Yes | mother | c.1912A>G | *SMPD1* | NM_000543.5 | p.Ala158Thr; c.472G>A | LP | na | Het | AD/RF | Positive |
| IP04779 | M | 65 | 56 | No | mother | c.366-35C>T | *SYNJ1* | NM_003895.3 | p.Ser1380Phe; c.4139C>T | VUS | na | Het | AR | Unknown |
| IP15836 | F | 78 | 60 | Yes | mother | c.1912A>G | *LRRK2* | NM_198578.4 | p.Gly2019Ser; c.6055G>A | P | na | Het | AD | Positive |
| IP16272 | M | 70 | 70 | Yes | father | c.1912A>G | *PINK1* | NM_032409.3 | p.Phe385Leu; c.1153T>C | LP | na | Het | AR | Unknown |
| IP16660 | F | 35 | 24 | No | - | c.1912A>G | *PARK2* | NM_004562.3 | p.Cys253Tyr; c.758G>A | LP | na | Het | AR | Positive |
|  |  |  |  |  |  |  |  |  | p.Arg275Trp; c.823C>T | LP | na | Het |  |  |
| IP16661 | M | 50 | 47 | No | - | c.1912A>G | *SMPD1* | NM_000543.5 | p.Gly492Ser; c.1474G>A | LP | na | Het | AD/RF | Positive |
| IP17056 | M | 45 | 41 | No | - | c.1912A>G | *GBA1* | NM_000157.4 | p.Leu483Pro; c.1448T>C | P | Severe | Het | AD/RF | Positive |

AAO: Age at Onset; M: Male; F:Female; LP: Likely Pathogenic; P: Pathogenic; B: Bening; VUS: Variant of Uncertain Significance; Het: Heterozygous; Hom: Homozygous; AR: Autosomic recessive; AD: Autosomic Dominant; RF: Risk Factor. Variants in *GBA1* gene were classified using the GBA1-PD browser [1].

**References:**

1. Parlar SC, Grenn FP, Kim JJ, Baluwendraat C, Gan-Or Z. Classification of GBA1 Variants in Parkinson’s Disease: The GBA1-PD Browser. *Movement Disorders*. 2023;38(3):489-495. doi:10.1002/mds.29314

**Table 2. Allele frequencies of rare variants detected in our local PD cohort and reference cohort.**

|  | **Cases** | | **Controls** | | | | | | | | |
| --- | --- | --- | --- | --- | --- | --- | --- | --- | --- | --- | --- |
|  | **Local PD** | | **CSVS** | | | **gnomAD (NFE)** | | | **gnomAD (global)** | | |
|  | **AC** | **AF** | **AC** | **AF** | ***p-value**** | **AC** | **AF** | ***p-value**** | **AC** | **AF** | ***p-value**** |
| **rs5908 (A>G)** | 46 | 0.020 | 71 | 0.015 | 1 | 24998 | 0.021 | 1 | 28704 | 0.018 | 1 |
| **rs144433856 (A>G)** | 23 | 0.010 | 28 | 0.015 | 1 | 2689 | 0.003 | **8.53e-06** | 3181 | 0.002 | **6.63e-09** |
| **rs377093901 (T>C)** | 6 | 0.003 | - | - | - | 1054 | 0.001 | 0.133 | 2779 | 0.002 | 1 |

AC: Allele Counts; AF: Allele Frequency; CSVS: Collaborative Spanish Variant Server; NFE: Non-Finish European. *Bonferroni correction is applied (n=8).
